# Developmental charts for children with osteogenesis imperfecta, type I (body height, body weight and BMI)

**DOI:** 10.1007/s00431-016-2839-y

**Published:** 2017-01-05

**Authors:** Krzysztof Graff, Malgorzata Syczewska

**Affiliations:** Department of Rehabilitation, The Children’s Memorial Health Institute, Al. Dzieci Polskich 20, 04-730 Warszawa, Poland

**Keywords:** Developmental charts, Osteogenesis imperfecta type I

## Abstract

Osteogenesis imperfecta (OI) is a rare genetic disorder of type I collagen. Type I is the most common, which is called a non-deforming type of OI, as in this condition, there are no major bone deformities. This type is characterised by blue sclera and vertebral fractures, leading to mild scoliosis. The body height of these patients is regarded as normal, or only slightly reduced, but there are no data proving this in the literature. The aim of this study is the preparation of the developmental charts of children with OI type I. The anthropometric data of 117 patients with osteogenesis imperfecta were used in this study (61 boys and 56 girls). All measurements were pooled together into one database (823 measurements in total). To overcome the problem of the limited number of data being available in certain age classes and gender groups, the method called reverse transformation was used. The body height of the youngest children, aged 2 and 3 years, is less than that of their healthy peers. Children between 4 and 7 years old catch up slightly, but at later ages, development slows down, and in adults, the median body height shows an SDS of −2.7.

*Conclusion*: These results show that children with type I OI are smaller from the beginning than their healthy counterparts, their development slows down from 8 years old, and, ultimately, their body height is impaired.

**Table:** 

**What is Known:**
• *The body height of patients with osteogenesis imperfecta type I is regarded as normal, or only slightly reduced, but in the known literature, there is no measurement data supporting this opinion.*
**What is New:**
• *Children with type I osteogenesis imperfecta are smaller from the beginning than their healthy counterparts, their development slows down from 8 years old and, ultimately, their final body height is impaired.* *• The developmental charts for the body height, body weight and BMI of children with type I osteogenesis imperfecta are shown.*

**Electronic supplementary material:**

The online version of this article (doi:10.1007/s00431-016-2839-y) contains supplementary material, which is available to authorized users.

## Introduction

Osteogenesis imperfecta (OI) is a rare genetic disorder of type I collagen. Its frequency is estimated as 1 in 20,000. There are several forms of this disease. Sillence [10] proposed a classification into four types, based on radiographic, clinical and genetic data; in some studies, even more are described [3, 7]. The most common is type I, which is called a non-deforming type of OI [7], as in this condition, there are no major bone deformities. This type is characterised by blue sclera and vertebral fractures, leading to mild scoliosis. Functional level and intellectual development, as well as life expectancy, are normal. As this type is the mildest of all OI types, the body height of these patients is acknowledged as normal or only slightly reduced [3]. But in the known literature, there is no measurement data supporting this opinion.

The assessment of body height and growth is a very important measure of the state of health of children, and paediatric charts are universally used [8]. Abnormal body height and growth (height velocity) could be symptoms of an underlying disease and need further diagnostics and attention [4]. This information on body height and growth should be easily accessible to paediatricians in monitoring their patients’ condition. Children with many genetic disorders experience affected growth and body height, so using the charts of healthy children is unreliable and could lead to the overlooking of some secondary problems also affecting body height and growth. In the literature, there are studies which prepared the growth charts of children with specific disorders to aid the detection of additional problems influencing growth patterns [2, 5, 11].

The aim of the present study is the preparation of the developmental charts of children with osteogenesis imperfecta, type I.

## Materials and methods

The anthropometric data of 117 patients with osteogenesis imperfecta type I according to the Sillence classification [10, 11] were used in this study (61 boys and 56 girls). The classification of OI patients into type I was retrospectively checked through the analysis of medical documentation and updated classification criteria. These children were being treated for their primary disease in The Children’s Memorial Health Institute and were being regularly measured. Children with comorbidities which could influence their body development were excluded from the database. Also, the measurements of patients who were undergoing surgical interventions for bone trauma (due to accidental fractures) were excluded from the database. The patients in our group neither required rodding nor had developed deformities which needed surgical correction. None of the patients had scoliosis or vertebral compression fractures. None of the patients had received bisphosphonates. All measurements were pooled together into one database (823 measurements in total). The number of measurements per patient varied from 1 to 35, with a median of 5 measurements per patient. The youngest patient who was measured was 4 months old; the oldest 22 years old. The data for patients older than 18 were treated as the time point of 18 years old. Because of the very limited number of data related to children less than 2 years of age, the charts were prepared for ages from 2 to 18 years. For children older than 1 year, body height was measured using an anthropometer. They stood with an upright posture looking straight ahead, with both legs and feet together, knees and legs straight, shoulders relaxed and arms by their sides. The accuracy of the measurement was within approximately 0.1 mm.

To overcome the problem of the limited number of data being available for certain age and gender groups, the method called reverse transformation, previously used to prepare the development charts of children with achondroplasia, was applied [2]. This method is described in Appendix 1. In the first step, the individual data of each patient were converted into a number. This number represented the difference between the raw score and the population mean in terms of the population’s standard deviation. The data (body height, body weight and BMI) of the healthy Polish population of children and adolescents (body height, body weight and BMI) were used as a reference population database [9].

A statistical analysis was performed using Statistica, v.10.0 (StatSoft), and regression curves were prepared using Matlab software. The Student *t* test was used for comparisons and the Spearman rank-correlation coefficient for checking the dependence between age and the analysed variables.

## Results

### Body height

As there was no statistically significant difference between boys and girls in normalised body height (Student’s *t* test *p* = 0.777), the data were pooled together. The correlation coefficient showed the dependence of normalised body height on age (*R* = −0.293, *p* < 0.005). Therefore, from the pooled database, the median, the upper and lower quartile and the 10th and 90th percentiles were calculated for the normalised body height (Tab. I in the Supplementary material). From these data, reverse transformation facilitated the calculation of the median, the upper and lower quartiles and the 10th and 90th percentiles of age groups for boys and girls separately (Table 1 and Table 2).

**Table 1:** The median, the upper and lower quartiles and the 10th and 90th percentiles of the age groups as related to body height for boys (in cm)

Age	Median	25%	75%	10%	90%
2	84.89204	81.2654	88.57112	79.6784	91.28696
3	93.31849	90.11833	95.70166	88.21315	97.0441
4	102.4787	95.37272	104.6983	91.20108	106.2073
5	109.0085	101.165	111.119	96.5615	113.117
6	114.8016	108.4026	116.994	104.1582	118.7166
7	120.8548	115.8753	123.0254	111.332	126.6855
8	123.5981	117.6521	127.74	112.5773	131.6797
9	126.202	122.2035	132.5985	114.0085	135.5795
10	131.7498	125.1858	139.9767	115.3027	142.9444
11	136.2484	131.5344	141.6709	122.0418	147.1256
12	140.8479	136.2939	147.5215	130.5008	154.9249
13	141.3209	134.1996	150.5995	124.079	154.9494
14	150.2725	139.4889	158.8425	132.4303	170.0083
15	161.5509	152.1941	165.0046	145.1971	168.6923
16	165.7032	157.7666	168.6334	149.3242	174.6581
17	167.5017	157.38	172.6904	147.5777	177.796
18	160.8012	154.5041	167.7682	151.2121	174.7862

**Table 2:** The median, the upper and lower quartiles and the 10th and 90th percentiles of the age groups as related to body height for girls (in cm)

Age	Median	25%	75%	10%	90%
2	82.29803	78.26405	86.39034	76.4988	89.41122
3	92.4794	89.2698	94.8696	87.359	96.216
4	100.8426	93.80456	103.0409	89.67284	104.5354
5	108.2572	100.2917	110.4005	95.61659	112.4296
6	113.1992	106.338	115.5499	101.7871	117.3969
7	119.7171	114.2041	122.1202	109.174	126.1725
8	124.2652	118.9434	127.9723	114.4013	131.448
9	124.4686	120.0024	131.5164	111.0336	134.8009
10	130.9693	124.5024	139.0745	114.7655	141.9983
11	137.0286	131.8095	143.0321	121.2999	149.0712
12	140.6173	135.8027	147.6729	129.678	155.5
13	146.3231	141.4024	152.7346	134.4091	155.7403
14	151.0475	143.5876	156.9761	138.7045	164.7005
15	154.4546	146.1994	157.5017	140.0262	160.7552
16	155.1322	148.1499	157.7101	140.7226	163.0103
17	156.0317	147.5097	160.4002	139.2568	164.6988
18	148.6516	142.6408	155.3019	139.4983	162.0009

The regression equation describing the regression curves for the median, the lower and upper quartiles and the 10th and 90th percentiles for body height was

$$ \mathrm{Body}\ \mathrm{height}=a1+a2*\mathrm{age}+a3*{\mathrm{age}}^{\wedge 2} $$

The constants a1, a2 and a3 for the regression equations for boys and girls are presented separately in Tables II and III in the Supplementary material.

**Fig. 1:**
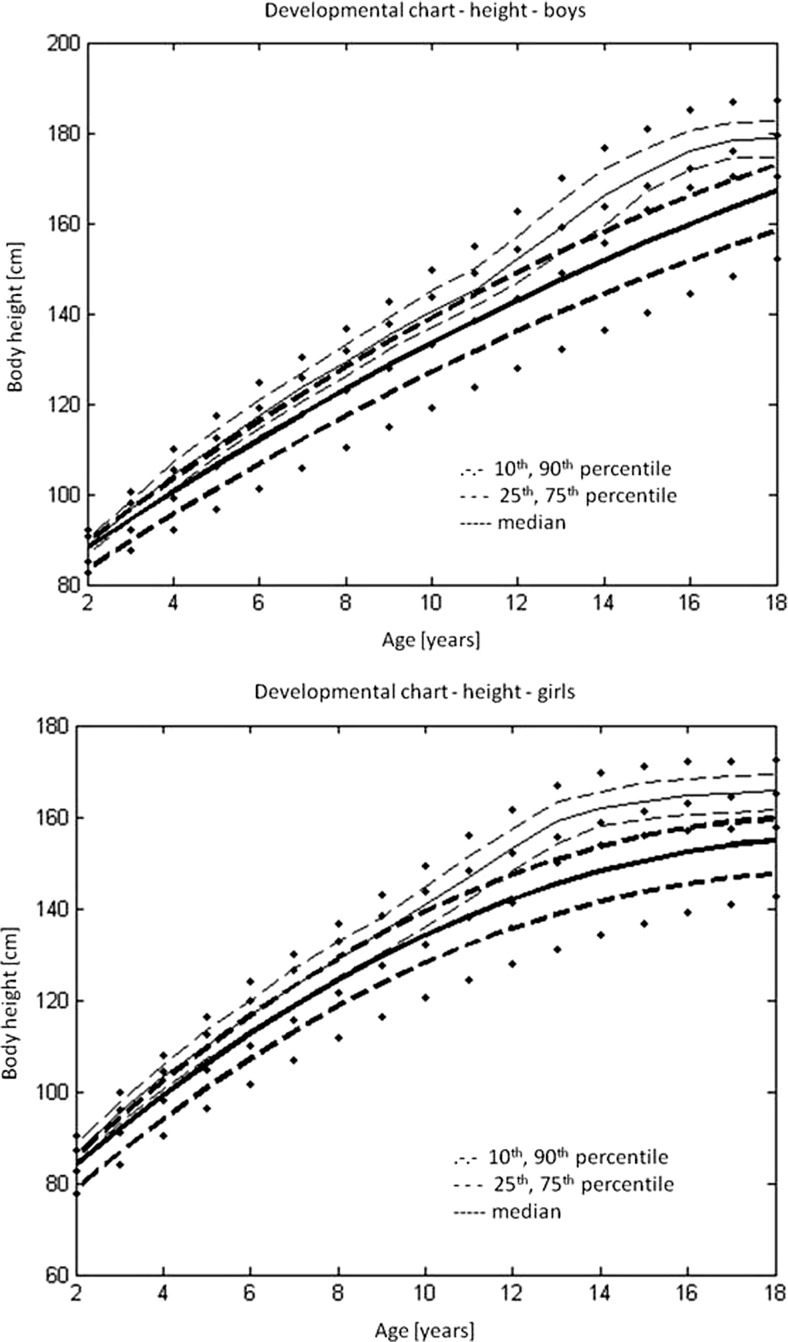
Developmental charts for the body height of children with type I osteogenesis imperfecta (*black*): **a** boys and **b** girls, against reference data on the healthy population (*grey*) [8]

Figure 1 shows the developmental charts of body height for children with type I osteogenesis imperfecta—(a) boys and (b) girls.

### Body weight

As there was no statistically significant difference between boys and girls in normalised body weight (Student *t* test *t* = −1.251, *p* = 0.211), the data were pooled together. The correlation coefficient showed the weak dependence of normalised body weight on age (*R* = −0.138, *p* < 0.005). Therefore, from the pooled database median, the upper and lower quartiles and 10th and 90th percentiles were calculated for the normalised body weight (Tab. IV in the Supplementary material). From these data, the reverse transformation facilitated the calculation of the median, the upper and lower quartiles and the 10th and 90th percentiles of the age groups for boys and girls separately (Table 3 and Table 4).

**Table 3:** The median, the upper and lower quartiles and the 10th and 90th percentiles of age groups as related to body mass for boys (in kg)

Age	Median	25%	75%	10%	90%
2	11.2688	9.6254	12.06575	8.78885	12.3842
3	13.83839	12.78503	14.8661	11.70089	15.89723
4	14.91476	12.71876	16.02008	11.35236	18.1624
5	17.09561	15.04583	19.0082	13.17359	22.65046
6	17.56953	15.39333	19.79006	13.66849	23.19944
7	20.51016	19.18152	22.45122	17.33388	26.60668
8	22.7997	17.85384	26.00109	15.23394	29.69451
9	24.16213	21.3046	28.92247	18.40066	34.05409
10	26.13356	21.99131	32.00372	17.71493	36.30377
11	29.20725	24.43875	38.59575	20.1735	47.67075
12	35.0093	29.39607	43.01018	24.99919	50.80383
13	34.73555	28.3001	44.5533	22.2846	52.80475
14	39.82025	30.3654	48.94635	23.22495	56.70485
15	48.12487	40.90735	52.60471	36.46899	65.41166
16	53.1368	45.40517	60.62117	43.10375	68.40986
17	54.84329	49.00226	59.67117	43.65459	71.2387
18	51.078	43.66276	58.07576	39.31898	75.05328

**Table 4:** The median, the upper and lower quartiles and the 10th and 90th percentiles of age groups as related to body mass for girls (in kg)

Age	Median	25%	75%	10%	90%
2	10.50776	8.93408	11.2709	8.13302	11.57584
3	13.51294	12.49038	14.5106	11.43794	15.51158
4	14.23985	12.30485	15.2138	11.10085	17.1015
5	16.4301	14.2203	18.492	12.2019	22.4186
6	17.65916	15.95276	19.40032	14.60028	22.07368
7	19.4056	17.5432	22.12645	14.9533	27.9513
8	23.2335	19.0152	25.96395	16.7807	29.11405
9	22.3015	19.5	26.9685	16.653	31.9995
10	26.1478	22.49905	31.3186	18.73215	35.10635
11	29.58709	25.00355	38.61143	20.90374	47.33443
12	33.7817	28.36783	41.49842	24.12711	49.01527
13	37.48655	32.7521	44.8763	28.3266	50.77975
14	42.0235	36.1996	47.6449	31.8013	52.4239
15	45.39462	40.0911	48.68646	36.82974	58.09716
16	45.6016	39.00004	51.99204	37.035	58.64232
17	44.33061	38.83434	48.87353	33.80231	58.1003
18	45	39.7034	49.9984	36.6007	62.1252

The regression equation describing the regression curves for the median, the lower and upper quartiles and 10th and 90th percentiles for body weight was

$$ \mathrm{Body}\ \mathrm{mass}=a1+a2*\mathrm{age}+a3*{\mathrm{age}}^{\wedge 2} $$

The constants a1, a2 and a3 for the regression equations for boys and girls are presented separately in Tables V and VI in the Supplementary material.

**Fig. 2:**
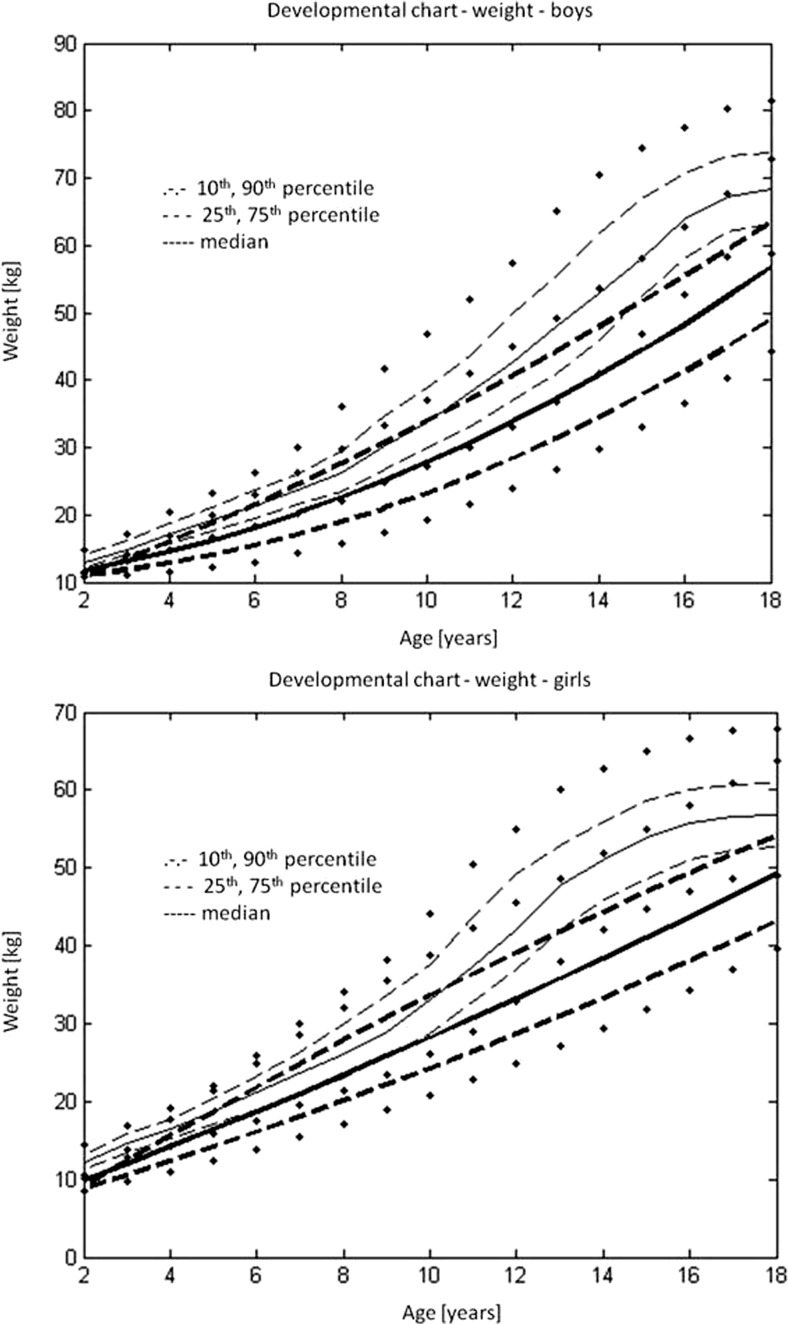
Developmental charts for the body mass of children with type I osteogenesis imperfecta (*black*): **a** boys and **b** girls, against reference data on the healthy population (*grey*) [8]

Figure 2 shows the developmental charts of body weight for children with type I osteogenesis imperfecta—(a) boys and (b) girls.

### BMI

As there was a statistically significant difference between boys and girls in normalised BMI (Student’s *t* test = −2.839, *p* = 0.005), the data could not be pooled together. The correlation coefficient in both gender groups showed no dependence of normalised BMI on age (*R* = 0.031, *p* > 0.05 for boys and *R* = 0.023, *p* > 0.005 for girls). As there was no dependence on the age median or the upper and lower quartiles, the 10th and 90th percentiles were calculated for normalised BMI separately for boys and girls (Table VII Supplementary material). From these data, reverse transformation facilitated the calculation of the median, the upper and lower quartiles and the 10th and 90th percentiles of the age groups for boys and girls separately (Tables VIII and IX Supplementary material).

The regression equation describing the regression curves for the median, the lower and upper quartiles and the 10th and 90th percentile BMI was

$$ BMI=a1+a2*\mathrm{age}+a3*{\mathrm{age}}^{\wedge 2} $$

The constants a1, a2 and a3 for the regression equations for boys and girls are presented separately in Tables X and XI in the Supplementary material.

**Fig. 3:**
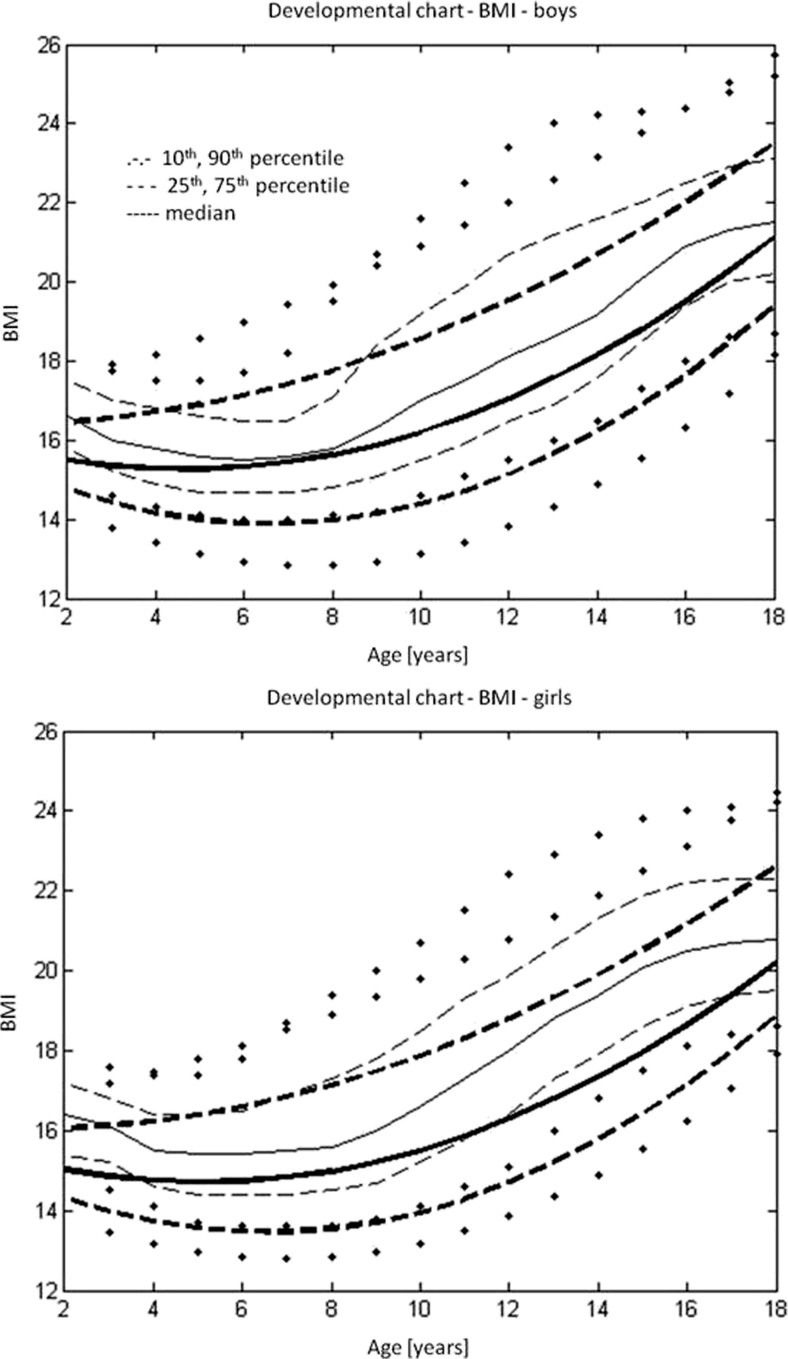
Developmental BMI charts for children with type I osteogenesis imperfecta (*black*): **a** boys and **b** girls, against reference data on the healthy population (*grey*) [8]

Figure 3 shows the developmental BMI charts for children with type I osteogenesis imperfecta—(a) boys and (b) girls.

## Discussion

Syndrome-specific developmental charts have proved to be helpful in medical practice [2, 8, 12]. Children with various syndromes could suffer from other comorbidities, which also negatively influence their development. Without the proper reference database, it is difficult to decide whether the impaired growth is being caused by primary disease or also by secondary diseases. In the case of rare diseases, it is difficult to compile enough measurements during the developmental process to be able to create proper developmental charts.

In this study, we used the so-called reversed transformation method developed for the construction of developmental charts for another rare disease—achondroplasia [2]. This method, together with regression equations, enabled the construction of developmental charts for boys and girls of 2 to 18 years with type I osteogenesis imperfecta. For rare diseases, it is difficult to collect enough data broken down by gender and age groups to construct developmental charts. Therefore, some alternatives must be found. In some cases, the data were gathered from various sources and literature [12]. Our method is an alternative which can be used when there is an insufficient number of subjects. This method has a drawback: as the curves are calculated using regression equations, the pubertal growth spurt is smoothed and does not stand out; this is the limitation of such a measure.

This type of OI is the mildest one—patients do not suffer from bone deformations, and their body height is regarded as normal, or only slightly reduced. Our results show that the body height of the youngest children, aged 2 or 3 years, is less than their healthy peers (the median is an SDS of −1.2 in the case of 2-year-olds, and an SDS of −0.9 in the case of 3-year-olds). Older children, between 4 and 7 years old, catch up slightly, and their median body height is around an SDS of −0.5, but at later ages, the development slows down, and in adults, the median body height exhibits an SDS of −2.7. These results are consistent with the results of the study of Aglan et al. [1]. Their study included 124 OI patients, but only 16 with OI type I, the age range being from 0.9 to 10.75 years. The mean height of these patients was an SDS of −0.426. Even the tallest OI type I patients (the 90th percentile) were smaller than their average healthy peers (an SDS of −0.5). The longitudinal study of Germain-Lee [6] on 36 patients with OI type I patients showed that their final body height was reduced in comparison with their healthy peers. These results show that children with type I OI are smaller from the beginning than their healthy counterparts, their development slows down from 8 years old and ultimately, their body height is impaired.

A similar trend can be observed in the case of body weight, inasmuch as the ratio between body height and body weight in type I OI patients is similar to that in healthy subjects. This fact is reflected in the body mass index (BMI) which is similar in OI patients to the BMI of healthy children and adolescents.

The patients in this study were classified into type I OI according to the Sillence classification [10, 11], which is based on the phenotype. As this was a retrospective study, in the case of the majority of the patients, there were no data on their genotype.

### Electronic supplementary material

Fig. S1 (DOCX 65 kb)

Fig. S2 (DOCX 62 kb)

Fig. S3 (DOCX 59 kb)

Table I (DOCX 11 kb)

Table II (DOCX 11 kb)

Table III (DOCX 11 kb)

Table IV (DOCX 11 kb)

Table V (DOCX 11 kb)

Table VI (DOCX 11 kb)

Table VII (DOCX 11 kb)

Table VIII (DOCX 12 kb)

Table IX (DOCX 12 kb)

Table X (DOCX 11 kb)

Table XI (DOCX 11 kb)

## Appendix 1

The individual data on each patient was standardised according to the following equation.

$$ {R}_{SDS}=\frac{R_i-{R}_N}{SD_N} $$

where

R_SDS_: the standardised results of the patient (body height, body weight or BMI)
R_i_: the individual measurement of the patient (body height, body weight or BMI, respectively)
R_N_: the mean value of the age- and gender-matched reference database for a given variable (body height, body weight or BMI, respectively)
SD_N_: the standard deviation for the age- and gender-matched reference database for a given variable (body height, body weight or BMI, respectively)

To create the developmental charts, data from Tables I, VI and XI (standardised values) were transformed according to the following equation:

$$ {X}_i={X}_{jk}+{W}_s\times {S}_{jk} $$

where

-X_i_: variable (body height, body weight or BMI) for the developmental chart
-X_jk_: the mean for the j-age and k-gender of the variable from the reference database
-W_s_: the standardised percentile for the given age of the variable (from Tables I, VI or XI, respectively)
-S_jk_: the standard deviation for the j-age and k-gender of the variable from the reference database

The reference database for this study was reference data on healthy Polish children and adolescents [8].

## Abbreviations

BMI: Body mass index
OI: Osteogenesis imperfecta
SDS: Standard deviation score
