# Supplementary material for: Developmental charts for children with osteogenesis imperfecta, type I (body height, body weight and BMI)
Source: Eur J Pediatr. 2017 Jan 5;176(3):311–6. doi: 10.1007/s00431-016-2839-y (PMC5321707; doi:10.1007/s00431-016-2839-y)

Fig.2. Developmental charts of body mass for children with type I osteogensis imperfect: a) boys, b) girls


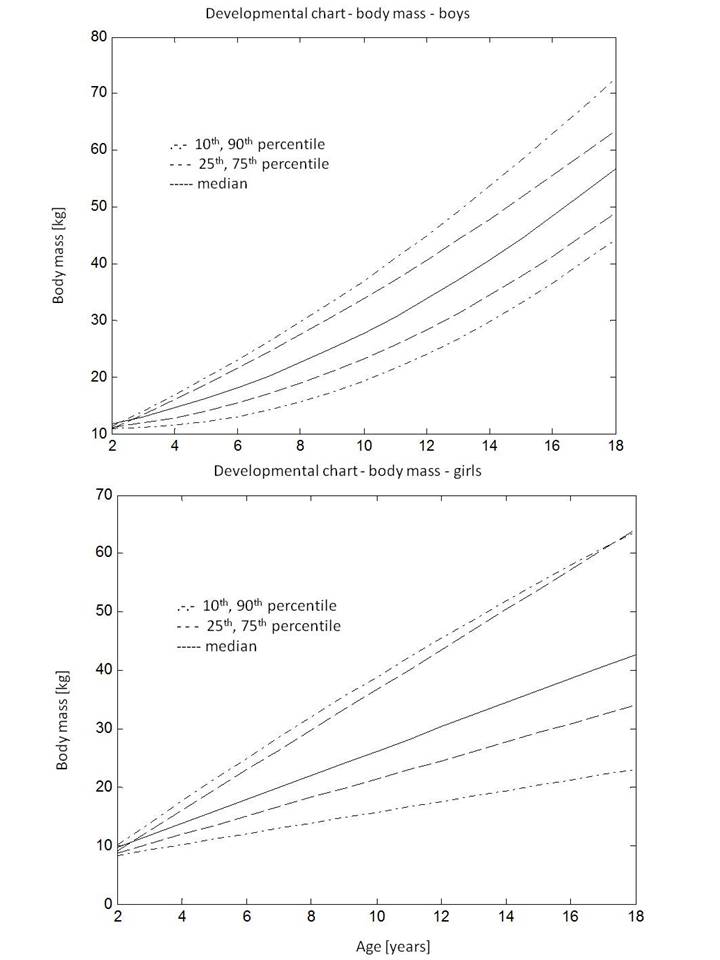

Supplement: Supplementary file 2 — (DOCX 62 kb) [file 431_2016_2839_MOESM2_ESM.docx]
