# Supplementary material for: Developmental charts for children with osteogenesis imperfecta, type I (body height, body weight and BMI)
Source: Eur J Pediatr. 2017 Jan 5;176(3):311–6. doi: 10.1007/s00431-016-2839-y (PMC5321707; doi:10.1007/s00431-016-2839-y)
Supplement: Supplementary file 4 — (DOCX 11 kb) [file 431_2016_2839_MOESM4_ESM.docx]

Table I. Median, upper and lower quartile, and 10 and 90th percentiles of age groups of the normalized body height.

| Age | N | Median | 25 % | 75 % | 10 % | 90 % |
| --- | --- | --- | --- | --- | --- | --- |
| 2 | 25 | -1.271 | -2.585 | 0.062 | -3.160 | 1.046 |
| 3 | 34 | -0.909 | -1.853 | -0.206 | -2.415 | 0.190 |
| 4 | 59 | -0.596 | -2.296 | -0.065 | -3.294 | 0.296 |
| 5 | 64 | -0.447 | -2.190 | 0.022 | -3.213 | 0.466 |
| 6 | 73 | -0.596 | -1.781 | -0.190 | -2.567 | 0.129 |
| 7 | 80 | -0.610 | -1.546 | -0.202 | -2.400 | 0.486 |
| 8 | 73 | -0.995 | -2.053 | -0.258 | -2.956 | 0.443 |
| 9 | 61 | -1.756 | -2.493 | -0.593 | -3.973 | -0.051 |
| 10 | 86 | -1.395 | -2.366 | -0.178 | -3.828 | 0.261 |
| 11 | 72 | -1.449 | -2.181 | -0.607 | -3.655 | 0.240 |
| 12 | 75 | -1.835 | -2.559 | -0.774 | -3.480 | 0.403 |
| 13 | 70 | -2.082 | -2.894 | -1.024 | -4.048 | -0.528 |
| 14 | 68 | -1.750 | -2.963 | -0.786 | -3.757 | 0.470 |
| 15 | 61 | -1.449 | -2.809 | -0.947 | -3.826 | -0.411 |
| 16 | 39 | -1.619 | -2.827 | -1.173 | -4.112 | -0.256 |
| 17 | 35 | -1.693 | -3.277 | -0.881 | -4.811 | -0.082 |
| 18 | 47 | -2.760 | -3.747 | -1.668 | -4.263 | -0568 |
