# Supplementary material for: Developmental charts for children with osteogenesis imperfecta, type I (body height, body weight and BMI)
Source: Eur J Pediatr. 2017 Jan 5;176(3):311–6. doi: 10.1007/s00431-016-2839-y (PMC5321707; doi:10.1007/s00431-016-2839-y)
Supplement: Supplementary file 7 — (DOCX 11 kb) [file 431_2016_2839_MOESM7_ESM.docx]

Table IV. Median, upper and lower quartile, and 10 and 90th percentiles of age groups of the normalized body mass.

| Age | N | Median | 25 % | 75 % | 10 % | 90 % |
| --- | --- | --- | --- | --- | --- | --- |
| 2 | 25 | -1.128 | -2.124 | -0.645 | -2.631 | -0.452 |
| 3 | 34 | -0.691 | -1.307 | -0.090 | -1.941 | 0.513 |
| 4 | 59 | -1.121 | -2.021 | -0.668 | -2.581 | 0.210 |
| 5 | 64 | -0.931 | -1.693 | -0.220 | -2.389 | 1.134 |
| 6 | 73 | -1.149 | -1.689 | -0.598 | -2.117 | 0.248 |
| 7 | 80 | -1.104 | -1.488 | -0.543 | -2.022 | 0.658 |
| 8 | 73 | -0.770 | -1.544 | -0.269 | -1.954 | 0.309 |
| 9 | 61 | -1.149 | -1.580 | -0.431 | -2.018 | 0.343 |
| 10 | 86 | -1.196 | -1.721 | -0.452 | -2.263 | 0.093 |
| 11 | 72 | -1.187 | -1.765 | 0.049 | -2.282 | 1.051 |
| 12 | 75 | -1.070 | -1.693 | -0.182 | -2.181 | 0.683 |
| 13 | 70 | -1.307 | -1.874 | -0.442 | -2.404 | 0.285 |
| 14 | 68 | -1.165 | -1.884 | -0.471 | -2.427 | 0.119 |
| 15 | 61 | -1.149 | -1.845 | -0.717 | -2.273 | 0.518 |
| 16 | 39 | -1.320 | -2.133 | -0.533 | -2.375 | 0.286 |
| 17 | 35 | -1.591 | -2.254 | -1.043 | -2.861 | 0.070 |
| 18 | 47 | -1.8 | -2.546 | -1.096 | -2.983 | 0.612 |
