# Supplementary material for: Developmental charts for children with osteogenesis imperfecta, type I (body height, body weight and BMI)
Source: Eur J Pediatr. 2017 Jan 5;176(3):311–6. doi: 10.1007/s00431-016-2839-y (PMC5321707; doi:10.1007/s00431-016-2839-y)
Supplement: Supplementary file 9 — (DOCX 11 kb) [file 431_2016_2839_MOESM9_ESM.docx]

Table VI. Constants (a1, a2, a3) for regression equations describing dependence of body mass from age for girls. The last two columns present correlation coefficient for the given regression curve and its p-level.

|  | a1 | a2 | a3 | R | p |
| --- | --- | --- | --- | --- | --- |
| median | 5,512 | 2,065 | 0,02 | 0,983 | <0.001 |
| 25 % | 5,617 | 1,578 | 0,028 | 0,982 | <0.001 |
| 75 % | 2,335 | 3,432 | -0,031 | 0,982 | <0.001 |
| 10 % | 6,49 | 0,919 | 0,051 | 0,970 | <0.001 |
| 90 % | 2,248 | 3,962 | -0,03 | 0,987 | <0.001 |
