# Supplementary material for: Developmental charts for children with osteogenesis imperfecta, type I (body height, body weight and BMI)
Source: Eur J Pediatr. 2017 Jan 5;176(3):311–6. doi: 10.1007/s00431-016-2839-y (PMC5321707; doi:10.1007/s00431-016-2839-y)
Supplement: Supplementary file 10 — (DOCX 11 kb) [file 431_2016_2839_MOESM10_ESM.docx]

Table VII. Median, upper and lower quartile, and 10 and 90th percentiles of the normalized BMI for boys and girls.

| sex | N | Median | 25 % | 75 % | 10 % | 90 % |
| --- | --- | --- | --- | --- | --- | --- |
| girls | 536 | -0.655 | -1.277 | 0.239 | -1.652 | 0.988 |
| boys | 524 | -0.470 | -1.135 | 0.403 | -1.591 | 1.252 |
