# Supplementary material for: Developmental charts for children with osteogenesis imperfecta, type I (body height, body weight and BMI)
Source: Eur J Pediatr. 2017 Jan 5;176(3):311–6. doi: 10.1007/s00431-016-2839-y (PMC5321707; doi:10.1007/s00431-016-2839-y)
Supplement: Supplementary file 11 — (DOCX 12 kb) [file 431_2016_2839_MOESM11_ESM.docx]

Table VIII. Median, upper and lower quartile, and 10 and 90th percentiles of age groups of the BMI for boys.

| Age | Median | 25% | 75% | 10% | 90% |
| --- | --- | --- | --- | --- | --- |
| 2 | 16,0274 | 14,9767 | 17,40674 | 14,25622 | 18,74816 |
| 3 | 15,4732 | 14,5156 | 16,73032 | 13,85896 | 17,95288 |
| 4 | 15,1622 | 14,0051 | 16,68122 | 13,21166 | 18,15848 |
| 5 | 15,0815 | 14,05075 | 16,43465 | 13,34395 | 17,7506 |
| 6 | 15,0216 | 13,8778 | 16,52316 | 13,09348 | 17,98344 |
| 7 | 15,0309 | 14,01345 | 16,36659 | 13,31577 | 17,66556 |
| 8 | 15,1546 | 13,2793 | 17,61646 | 11,99338 | 20,01064 |
| 9 | 15,8263 | 14,02415 | 18,19213 | 12,78839 | 20,49292 |
| 10 | 16,3417 | 14,41985 | 18,86467 | 13,10201 | 21,31828 |
| 11 | 16,5746 | 14,6993 | 19,03646 | 13,41338 | 21,43064 |
| 12 | 17,6219 | 15,47395 | 20,44169 | 14,00107 | 23,18396 |
| 13 | 17,8272 | 15,6726 | 20,65572 | 14,19516 | 23,40648 |
| 14 | 18,259 | 16,0645 | 21,1399 | 14,5597 | 23,9416 |
| 15 | 19,0334 | 17,1847 | 21,46034 | 15,91702 | 23,82056 |
| 16 | 19,8721 | 18,16305 | 22,11571 | 16,99113 | 24,29764 |
| 17 | 20,4656 | 18,7898 | 22,66556 | 17,64068 | 24,80504 |
| 18 | 20,5677 | 19,3232 | 23,7678 | 18,2345 | 27,7689 |
