# Supplementary material for: Developmental charts for children with osteogenesis imperfecta, type I (body height, body weight and BMI)
Source: Eur J Pediatr. 2017 Jan 5;176(3):311–6. doi: 10.1007/s00431-016-2839-y (PMC5321707; doi:10.1007/s00431-016-2839-y)
Supplement: Supplementary file 12 — (DOCX 12 kb) [file 431_2016_2839_MOESM12_ESM.docx]

Table IX. Median, upper and lower quartile, and 10 and 90th percentiles of age groups of the BMI for girls.

| Age | Median | 25% | 75% | 10% | 90% |
| --- | --- | --- | --- | --- | --- |
| 2 | 15,4227 | 14,39018 | 16,90674 | 13,76768 | 18,15008 |
| 3 | 15,1816 | 14,38544 | 16,32592 | 13,90544 | 17,28464 |
| 4 | 14,62335 | 13,73389 | 15,90177 | 13,19764 | 16,97284 |
| 5 | 14,3803 | 13,29802 | 15,93586 | 12,64552 | 17,23912 |
| 6 | 14,60095 | 13,66173 | 15,95089 | 13,09548 | 17,08188 |
| 7 | 14,6866 | 13,26844 | 16,72492 | 12,41344 | 18,43264 |
| 8 | 14,64975 | 13,06365 | 16,92945 | 12,1074 | 18,8394 |
| 9 | 14,4608 | 12,81872 | 16,82096 | 11,82872 | 18,79832 |
| 10 | 15,6353 | 13,93102 | 18,08486 | 13,9639 | 20,13712 |
| 11 | 16,02255 | 14,28717 | 18,51681 | 13,24092 | 20,60652 |
| 12 | 16,33605 | 14,41407 | 19,09851 | 13,25532 | 21,41292 |
| 13 | 17,29875 | 15,58825 | 19,75725 | 14,557 | 21,817 |
| 14 | 17,90735 | 16,27149 | 20,25857 | 15,28524 | 22,22844 |
| 15 | 18,6725 | 17,1175 | 20,9075 | 16,18 | 22,78 |
| 16 | 19,077 | 17,4598 | 21,4014 | 16,4848 | 23,3488 |
| 17 | 19,18085 | 17,35839 | 21,80027 | 16,25964 | 23,99484 |
| 18 | 19,5118 | 17,99412 | 21,69316 | 17,07912 | 23,52072 |
