# Supplementary material for: Developmental charts for children with osteogenesis imperfecta, type I (body height, body weight and BMI)
Source: Eur J Pediatr. 2017 Jan 5;176(3):311–6. doi: 10.1007/s00431-016-2839-y (PMC5321707; doi:10.1007/s00431-016-2839-y)
Supplement: Supplementary file 13 — (DOCX 11 kb) [file 431_2016_2839_MOESM13_ESM.docx]

Table X. Constants (a1, a2, a3) for regression equations describing dependence of BMI from age for boys. The last two columns present correlation coefficient for the given regression curve and its p-level.

|  | a1 | a2 | a3 | R | p |
| --- | --- | --- | --- | --- | --- |
| median | 15.980 | -0.308 | 0,033 | 0,983 | <0.001 |
| 25 % | 15.697 | -0.551 | 0.042 | 0,981 | <0.001 |
| 75 % | 16.353 | 0.001 | 0.022 | 0,972 | <0.001 |
| 10 % | 15.502 | -0.717 | 0.048 | 0,970 | <0.001 |
| 90 % | 16.715 | 0.320 | 0.010 | 0,958 | <0.001 |
